# NBAS mutations cause acute liver failure: when acetaminophen is not a culprit

**DOI:** 10.1186/s13052-017-0406-4

**Published:** 2017-09-25

**Authors:** Pier Luigi Calvo, Francesco Tandoi, Tobias B. Haak, Andrea Brunati, Michele Pinon, Dominic Dell Olio, Renato Romagnoli, Marco Spada

**Affiliations:** 1 Pediatric Gastroenterology Unit, Regina Margherita Children’s Hospital, Azienda Ospedaliero-Universitaria Città della Salute e della Scienza di Torino, University of Turin, Piazza Polonia 94, 10126 Torino, Italy; 2 Liver Transplantation Center, Azienda Ospedaliero-Universitaria Città della Salute e della Scienza di Torino, University of Turin, Torino, Italy; 3 Institute of Human Genetics, Technische Universität München, 81675 Munich, Germany; 4 Regional Transplant Center, Azienda Ospedaliero-Universitaria Città della Salute e della Scienza di Torino, University of Turin, Torino, Italy; 5 Department of Pediatrics, Regina Margherita Children’s Hospital, Azienda Ospedaliero-Universitaria Città della Salute e della Scienza di Torino, University of Turin, Torino, Italy; 6 Institute of Human Genetics, Helmholtz Zentrum München, 85764 Neuherberg, Germany

**Keywords:** Acute liver failure, Acetaminophen toxicity, N-Acetylcysteine, *NBAS* mutation, Liver Transplantation

## Abstract

**Background:**

Pediatric acute-liver-failure due to acetaminophen (APAP) administration at therapeutic dosage is rare, while viral infections and metabolic defects are the prevalent causes. Yet, as acetaminophen is routinely used in febrile illnesses, it may be mistakenly held responsible for the acute liver damage.

**Case presentation:**

An 11 month old boy had been on acetaminophen for 10 days (total dose 720 mg = 72 mg/kg) when he developed acute-liver-failure with encephalopathy. As he rapidly improved on N-acetylcysteine (NAC) infusion, it was concluded that chronic acetaminophen administration in an infant had lead to acute-liver-failure even at therapeutic doses, that N-acetylcysteine infusion had been life-saving and should be immediately started in similar circumstances. The child, however, had two further episodes of acute liver damage over a 34-month period, without having been given acetaminophen, as the parents carefully avoided using it. His clinical, laboratory and radiological findings between the acute episodes were unremarkable. His features and skeletal surveys were not suggestive of a syndromic condition. He then went on to suffer another episode of acute-liver-failure with multi-organ failure, necessitating an urgent liver transplant. All efforts to come to a diagnosis for the causes of his recurrent episodes of liver failure had been unsuccessful, until a biallelic mutation in the *NBAS* gene was reported to be associated with recurrent acute-liver-failure in children. The boy’s DNA analysis revealed compound heterozygous pathogenic mutations in the *NBAS* gene*.* Liver failure episodes in these patients are triggered and worsened by fever, most likely due to thermal susceptibility of hepatocytes, hence APAP, rather than being a culprit, is part of the supportive treatment.

**Conclusions:**

We suggest that, in acute-liver-failure with a history of acetaminophen exposure at therapeutic dosage, clinicians should not be contented with administering NAC, but should consider an alternative etiology, above all if the episodes are recurrent, and actively start supportive and antipyretic treatment while seeking the advice of a specialist unit.

## Background

Acute liver failure (ALF) is a rare syndrome, characterized by rapid onset of severe hepatic dysfunction and hepatocellular damage in the absence of pre-existent liver disease [1]. In children, it is a devastating illness that still carries a high risk of mortality. In up to 50% of cases a cause cannot be found despite thorough investigation [2].

The syndrome may have diverse etiologies that manifest wide geographic and age differences. Etiology is an important factor in determining outcome. Patients with acute acetaminophen (N-acetyl-p-aminophenol = APAP) toxicity would be expected to have a relatively good prognosis, given the known pathobiology and the availability of a treatment for this condition. Yet, individual patients with APAP-induced ALF may die [3].

APAP is a widely-used medication. It is available without prescription and is a trusted remedy in most home medicine cabinets. APAP is considered safe when taken at the recommended dose (10–15 mg/kg) and frequency (fewer than 5 doses/day) for a total recommended daily dose of 75 mg/kg per day [4]. Yet APAP toxicity is the commonest cause for liver failure and liver transplantation in the Western world [5].

Pediatric acute-liver-failure due to chronic acetaminophen administration at therapeutic dosage is rare [6]. In one instance, such association was mistakenly reported.

## Case Presentation

The case of an 11-month-old boy (10 kg), now 7, was published in 2011 [7]. He had presented in discrete general health with a 10-day history of fever and vomiting. Investigations showed significantly elevated liver enzymes (LFT) (AST 11,735 IU/L [normal range: < 41 IU/L], ALT 6611 IU/L [normal range: < 41 IU/L]) and γGT 286 IU/L [normal range: < 71 IU/L]), normal total serum bilirubin and normal clotting. His LFT values remained unchanged the following day, while ammonium concentration was high (266 μg/dL [normal range: < 125 μg/dL]), total bilirubin increased significantly (2.18 mg/dL, direct 1.51 mg/dL), the international normalized ratio (INR) rose to 2.07 [normal range: < 1.20] and did not respond to parenteral vitamin K administration. He became lethargic with a normal electroencephalogram (EEG) and the clinical picture suggested acute-liver-failure (ALF).

His pediatrician had been treating his fever with APAP suppositories at 125 mg (12.5 mg/kg) every 6 h for 3 days, followed by oral administration at 120 mg (12 mg/kg) every 4 h for 7 days, for a total dose of 720 mg (72 mg/kg) over 10 days. His serum APAP concentration 9 h after the last dose was 7.2 mg/mL (reference range: 10.0–30.0 mg/mL). He was given *N*-acetylcysteine (NAC) infusion at a dose of 150 mg/kg in 5% dextrose in 90 min and then 150 mg/kg/day with rapid improvement of his LFT on the first day and normalization of the INR within 5 days. Five and 10 months later, two further febrile episodes lasting less than 24 h with only a slight ALT alteration (102 and 65 IU/L respectively), were not treated with APAP. An exhaustive etiological screening for pediatric ALF was inconclusive and as the child had remained in good health for 12 months, the clinicians reporting his case presumed that his ALF had been induced by chronic APAP administration at therapeutic dosage and that NAC treatment had been “life-saving” [1, 4, 5].

Twenty-three months after his first ALF episode, when he was 34 months old, he re-presented with lethargy after 6 h of fever and recurrent vomiting. There was a marked alteration in AST (15,487 IU/L), ALT (10,088 IU/L), normal γGT (35 IU/L) and total bilirubin, an increased INR (3.27) and a high ammonium concentration (453 μg/dL). EEG showed diffuse cerebral suffering. Supportive treatment and NAC infusion were commenced immediately, but the encephalopathy and liver dysfunction worsened dramatically. Within 36 h the INR rose to 5.06, AST and ALT were 20,070 and 12,830 IU/L respectively, LDH was 22,392 IU/L and ammonium 479 μg/dL. He was listed for urgent liver transplantation, but after 24 h his LFT and clotting started improving and he was taken off the transplant list.

No etiological diagnosis was reached despite an exhaustive infectious and metabolic screening **(**See Table 1 **).** He showed no syndromic features and there was no family history of liver involvement. He was repeatedly investigated for mitochondrial disorders as these ALFs can be stress-induced, without signs or symptoms of neuromuscular disease, but liver and muscle biopsies were first postponed, due to an upper respiratory tract infection not associated with clotting or LFTs alterations and then cancelled due to withdrawal of the parental consent.

**Table 1:** Results of the diagnostic workup

Tests	Result	Reference Range
Serum APAP concentration (9 h after the last administration)	7.2 mg/mL	10.0–30.0 mg/mL
PCR for CMV/EBV/HHV6/HHV1–2/ Adenovirus/Parvovirus B19/Paramixovirus/HBV/HCV/HIV	Negative	Negative
Serology for HAV/HBV/HIV/HHV6/Parvovirus B19/HHV1–2/Paramixovirus/Enterovirus	Negative	Negative
Plasma aminoacidogram, acylcarnitine profile, urinary organic acids, orotic acid, succinylacetone and δ-aminolevulinic acid measurements	N.A.D.	N.A.D
Ammonium	266–479 μg/dL during crisis, repeatedly normal between crisis	< 125 μg/dL
Serum transferrin isoelectrofocusing	Normal profile	
Ceruloplasmin	25 ng/ml	>20 ng/ml
Urinary copper level	10 μg/die	<40 μg/die
Autoantibody (ANA, ASMA, LKM AMA)	Negative	Negative
CPK	81UI/L	25–190 UI/L
Colesterol Trygliceride	130 mg/dL 60 mg/dL	<105 mg/dL 150–200 mg/dL
Glicemia	90 mg/dL	70–100 mg/dL
IgG;IgA;IgM	1120 mg/dL;92 mg/dL;112 mg/dL	(605–1230 mg/dL) (30–198 mg/dL) (45–200 mg/dL)
Lymphocyte subpopulations	Normal for age	Normal for age
EEG	Slight diffuse cerebral suffering during latest crisis, otherwise normal	Normal
Brain Magnetic Resonance Imaging	Normal for age	Normal for age
Echocardiogram	N.A.D.	N.A.D.
Muscle biopsy	Normal respiratory chain enzyme activity	Normal respiratory chain enzyme activity
Renal tubular function	N.A.D.	N.A.D.
DGUOK/POLG sequencing	No mutations	
Exome sequencing	Two heterozygous variant (frameshift and missense) in NBAS	Absent from >120.000 control alleles of the ExAC browser an in about 7500 in-house control exomes (Munich Exome Server)
Rx skeletal	N.AD.	N.A.D.
Ophthalmologic evaluation	N.A.D.	N.A.D.

*NAD* (nihil abnormal detected)

Eleven months later, aged 3.7 years, he was urgently re-admitted with a catastrophic ALF episode after 3 days of sore throat and untreated fever. There was multiorgan involvement, renal dysfunction requiring dialysis and lung complications which impaired respiratory exchanges. A reduced liver (segments I to IV), from a 45-year-old small-sized deceased male donor was urgently transplanted. He made a full recovery and he was discharged on the 59^th^ postoperative day.

The explanted liver histology showed massive pan-lobular necrosis, coagulative central necrosis, edematous portal spaces with scanty portal mononuclear and granulocytic infiltration. Some hepatocytes were still preserved in acinar zone 1 and showed cytoplasmic vacuolization and degeneration. Diffuse necrosis precluded metabolic investigations on the explanted liver tissue. Although neurological signs were absent, a muscle biopsy, performed 6 months after transplantation, showed normal respiratory chain enzyme activities. Genetic testing for deoxyguanosine kinase (DGUOK), DNA polymerase γ (POLG) mutations and other genes commonly involved in mitochondrial DNA depletion [8–11], showed no pathogenic mutations [7, 12, 13].

The child, now 8, shows normal growth and neurodevelopment at his routine follow-up visits.

DNA analysis revealed compound heterozygous pathogenic mutations in *NBAS* (NM_015909.3): c.[809G > C];[2926del], p.[Gly270Ala];[Ser976Profs*16]. Both variants were absent from 7500 in-house exomes from individuals with unrelated phenotypes and about 120,000 alleles of the Exome Aggregation Consortium (ExAC) Server (Cambridge, MA [03/2016]). The paternally inherited c.809G > C mutation affects an evolutionarily highly conserved glycine and is predicted as functionally relevant in silico (PolyPhen 2: probably damaging (0.998); SIFT score: 0; CADD score 19.45). The maternally inherited 1 bp deletion c.2926del causes a frameshift and predicts a prematurely truncated protein, missing more than 50% of wild-type polypeptide sequences.

## Discussion and conclusions

Although it is rare, pediatric ALF is a devastating event, accounting for 10–15% of all pediatric liver transplants. Pediatric ALF is defined as a coagulopathy, with an INR ≥1.5 not correctable by parenteral vitamin K administration, in the presence of grade III or IV hepatic encephalopathy (HE), or an INR ≥2.0, regardless of the presence/absence of clinical HE, and biochemical evidence of acute hepatocellular injury in children with no previous evidence of liver disease [1, 2, 14]. Metabolic disorders and viral infections are the prevalent causes of ALF in neonates and infants, whilst drug-induced hepatotoxicity and autoimmune hepatitis are more commonly identified in older children; the etiology remains undetermined in up to 50% of cases [2, 8–11, 14]. RALF, with complete clinical and biochemical recovery between the critical events, is even rarer. Reported etiologies include viruses, autoimmune hepatitis, metabolic disorders affecting the mitochondrial respiratory chain, the long-chain fatty acid oxidation pathway or the carnitine cycle, dihydrolipoamide dehydrogenase (E3) deficiency (OMIM: #246900) and Wolcott-Rallison syndrome (OMIM: #226980).

Early identification of the cause of ALF is fundamental as it is sometimes reversible if specific therapy is started. While the etiological screening is carried out, supportive treatment with restricted hydration, broad spectrum antibiotics, antivirals and antimycotics and adequate caloric support should be started immediately [8].

If there is a suspicion that ALF may be APAP-induced, NAC is the elective antidote [9–11, 15], making it crucial to ascertain whether APAP administration may be responsible for the ALF. Yet, in some metabolic disorders, ALF is triggered by fever and/or viral-infection [16] and almost all patients have a history of APAP assumption due to antipyretic treatment. The recommended APAP oral dose in children under six is 10 to 15 mg/kg/dose, with a maximum dose of 90 mg/kg/day. A daily dose of 150 mg/kg or higher has been reported to be hepatotoxic [17, 18] whereas < 75 mg/kg/die has been reported to be safe [4, 9, 18] even if anecdotic reports have suggested that younger children may develop toxicity after repeated therapeutic doses of APAP [9, 17, 18].

If this were the case, then NAC treatment should be started without delay and it may still be useful 48 h after APAP ingestion [8, 10]. Approved national triage guidelines for repeated supratherapeutic APAP ingestion recommend that children younger than 6 years should be referred for emergency evaluation if they have ingested 200 mg/kg or more over a single 24-h period, 150 mg/kg per 24-h period for the preceding 48 h, or 100 mg/kg per 24-h period for the preceding 72 h or longer.

NAC is a thiol-group donor that assists with replenishing of liver stores of glutathione (GSH), the primary antioxidant in hepatocytes and represents the specific antidote for paracetamol poisoning. On the efficacy of NAC infusion in non-APAP-induced ALF, the two largest multicenter, randomized, double-blind, placebo controlled studies in adults [19] and in children [20] reached divergent conclusions. One proposed mechanism of hepatocyte damage in ALF is the production of reactive oxygen species (ROS). Upon donating an electron to unstable ROS, two GSH react together to form glutathione disulfide. Increasing mitochondrial GSH can be an effective strategy to prevent mitochondrial oxidative stress and pro-inflammatory cytokines generation [21]**.** A recent meta-analysis concluded that NAC use for non-APAP-induced ALF is safe, it can prolong patients’ survival with native liver without transplantation, especially in the milder grades of hepatic encephalopathy (I or II), but it does not affect the overall survival [22]. Nonetheless, NAC infusion is part of the initial supportive treatment in pediatric ALF while investigations are ongoing [8]. There is a feeling amongst operators that it may be useful in ALF caused by other chemicals (drugs, mushroom poisoning) [23] and there is experimental evidence of its role in protecting mitochondria from damage, even if the value of NAC use in less severe forms of drug-induced liver injury is still being assessed [24].

The clinical presentation of this 11-month old child was suggestive of ALF due to repeated therapeutic doses of acetaminophen [7]. However, the child had two further episodes of acute liver damage over a 34-month period without having taken APAP, whilst there were no clinical or laboratory signs of liver disease between the episodes. Temperature was the likely triggering factor in all three RALF episodes.

A report that mutations in the *neuroblastoma amplified sequence* gene (*NBAS*; OMIM #616483) can cause fever-triggered recurrent liver failure (RALF) in children was published in 2015 [16]. This mutation may also be associated with a multisystemic phenotype of short stature, skeletal dysplasia, optic atrophy and immunological abnormalities (Pelger-Huet), with normal motor and cognitive development, resembling the SOPH syndrome occurring in the Yakut population [25]. Our patient, however, had none of these features and skeletal surveys, bone densitometry, MRI, neurological, eye examination and growth evaluation showed no syndromic peculiarities [16, 25–28].

NBAS in zebra fish, nematodes (C.elegans) and humans has been shown to act in concert with core factors of the nonsense-mediated mRNA decay (NMD) pathway to co-regulate a large number of endogenous RNA targets [29]. NMD modulates cellular stress response pathways and membrane trafficking. Lack of NBAS function could affect its role in NMD, its activity as a component of the syntaxin 18 complex or compromise yet unreported cellular functions. Thermal susceptibility of the syntaxin 18 complex is the basis of fever dependency of ALF episodes. Depletion of NBAS leads to a significant upregulation of the matrix G1a protein (MGP), responsible for bone formation. Thus, an increased MGP activity would be compatible with the short stature and associated bone defects in the SOPH syndrome [25]. The phenotype and medical history of 14 individuals with NBAS deficiency showed that RALF may present from 4 months to 6.7 years, but is not restricted to childhood, and that ALF crisis, as in our case, could be fatal without a liver transplant [28, 30]. Characteristically, these patients presented with recurrent vomiting and increasing lethargy 1 or 2 days after the onset of fever. Syndromic SOPH-like features and stunted linear growth have been reported in up to 77% of the 22 children with pathological NBAS mutations described to date, while liver cytolytic episodes, which were absent in the original 33 SOPH cases, were present in all 22, but did not necessarily evolve into ALF [31]. Intermediate phenotypes have recently been described [32] Antipyretic therapy and anabolic support, including high glucose and parenteral lipids, effectively improve the liver crisis [8, 16, 28, 30]. As our patient had these characteristics, we tested him for a mutation in the *NBAS* gene, with positive results. Not having administered APAP, which was mistakenly considered the culprit of his liver damage, might, in fact, have been one of the factors that led to the catastrophic evolution of the last episode.

In conclusion, genetic defects of the energy pathways (mitochondrial diseases have a prevalence of approximately 1 in 5000), should be part of the differential diagnosis in individuals presenting with ALF in isolation or as part of a multisystemic disease presentation, especially if drugs are involved [33].

Our case raises the suspicion that APAP assumption at therapeutic doses, in some other reports of an association between chronic administration of the drug and ALF in infants with APAP levels within the normal range on admission, may have been a red herring, leading to a hasty diagnosis and an incorrect management of the ALF episode.

## Abbreviations

ALF: acute liver failure
APAP: acetaminophen
DGUOK: deoxyguanosine kinase
EEG: electroencephalogram
GSH: glutathione
HE: hepatic encephalopathy
INR: international normalized ratio
LFT: liver function tests
MGP: matrix G1a protein
NAC: N-acetylcysteine
NBAS: neuroblastoma amplified sequence
NMD: nonsense-mediated mRNA decay
POLG: DNA polymerase γ
RALF: recurrent acute liver failure
ROS: reactive oxygen species
